# Evaluation of drugs and risk factors requiring therapeutic drug monitoring in the Neonatal Intensive Care Unit: a retrospective study

**DOI:** 10.1186/s12887-026-06929-w

**Published:** 2026-05-07

**Authors:** Yunus Emre Ayhan, Şükran Yıldırım, Ercan Tutak, Avidan Kızılelma Yiğit, Çiğdem Kırmacı, Adem Karbuz

**Affiliations:** 1 Clinical Pharmacy, Prof. Dr. Cemil Taşcıoğlu City Hospital, Istanbul, Türkiye; 2 Neonatal Intensive Care Unit, Prof. Dr. Cemil Taşcıoğlu City Hospital, Istanbul, Türkiye; 3 Department of Pediatric Infectious Diseases, Prof. Dr. Cemil Taşcıoğlu City Hospital, Istanbul, Türkiye

**Keywords:** Neonatal intensive care unit, Therapeutic drug monitoring, Clinical pharmacist, Neonatalogist, Risk factors, Gentamicin

## Abstract

**Background:**

Neonates in the Neonatal Intensive Care Units (NICU) often receive medications requiring therapeutic drug monitoring (TDM) due to their immature organ function and the narrow therapeutic windows of these drugs. Despite its critical role in optimizing dosing and minimizing toxicity, data on the prevalence and risk factors associated with TDM-requiring drugs in neonates is limited. This study aims to estimate the potential burden of TDM-requiring drug use in a NICU where routine TDM is not implemented and to identify key clinical and demographic factors associated with their use.

**Methods:**

A retrospective, descriptive study was conducted on neonates admitted to a NICU between January 1, 2019, and July 1, 2024. Patients who received at least one TDM-requiring drug were included. The drugs included in the patients’ treatments that required TDM were accepted as antibiotics (vancomycin, gentamicin, amikacin), antiepileptics (phenytoin, phenobarbital, carbamazepine, valproic acid, levetiracetam) and digoxin. Data were analyzed using chi-square, Mann-Whitney U, Kruskal-Wallis, and binary logistic regression tests.

**Results:**

Among 3754 neonates, 1404 (37.4%) received TDM-requiring drugs, with 1375 meeting inclusion criteria. The most commonly TDM-requiring drugs were gentamicin (62.5%), vancomycin (13.6%), and amikacin (11.5%). Risk factors significantly associated with TDM included mechanical ventilation (OR = 4.3, 95% CI: 3.2–5.5), total parenteral nutrition (OR = 4.0, 95% CI: 3.1–5.2), and NICU hospitalization ≥ 10 days (OR = 7.3, 95% CI: 5.4–9.9).

**Conclusion:**

A substantial proportion of neonates in the NICU are exposed to medications requiring TDM. In a setting where routine TDM is not implemented, this finding highlights a potential unmet need for monitoring. Mechanical ventilation, prolonged NICU hospitalization, and total parenteral nutrition were identified as key risk factors. Identifying these high-risk groups may support targeted monitoring strategies and optimize resource allocation in NICUs where TDM is not routinely available.

**Trial registration:**

Not applicable.

## Background

The neonatal population undergoes a progressive maturation process regarding metabolism and organ function after birth. Therefore, “children are not small adults” should be a cornerstone of pharmacological approaches in this population [1]. In Neonatal Intensive Care Units (NICUs), approximately 65% of the drugs used for treatment are off-label, highlighting the need for optimization of dosage management. Therapeutic drug monitoring (TDM) is critical in optimizing drug exposure to ensure clinical efficacy and prevent toxicity [2]. Consequently, TDM has become indispensable in NICUs, where newborns often receive complex and multi-drug treatments [3].

Drug response in neonates is highly variable due to inter-individual differences influenced by a range of factors [4]. Key pharmacokinetic parameters such as volume of distribution (Vd), clearance (Cl), and absorption rates significantly affect how drugs are metabolized and distributed in this fragile population [5]. Currently, neonate dosage regimens are often extrapolated from adult data using linear weight-based scaling, which is suboptimal. The physiological immaturity of neonates increases the risk of therapeutic failures and adverse effects, necessitating personalized pharmacotherapy [6]. The primary purpose of TDM is to measure plasma drug concentrations to personalize drug dosages and minimize risks [7]. While not all drugs require TDM, it is vital for certain high-risk medications, making it a critical component of neonatal care [8].

Two primary drug groups requiring TDM in neonates are antimicrobials and antiepileptic drugs (AEDs). Antibiotics such as gentamicin, ampicillin, and vancomycin are commonly used to improve clinical outcomes in NICUs [9]. These drugs are among the most frequently prescribed in European NICUs [10, 11]. However, ethical concerns about enrolling neonates in randomized controlled trials and limited neonatal-specific pharmacological data hinder the development of precise dosing regimens [12, 13]. Among AEDs, phenobarbital and phenytoin are still the most commonly used, while the increasing use of levetiracetam—reported to have no neurotoxic effects—has gained attention in recent years [14–16].

Despite the recognized importance of TDM in neonatal care, there is a lack of data evaluating the real-world burden of TDM-requiring drug use, particularly in settings where routine TDM is not implemented. Moreover, limited evidence exists on which neonatal subgroups are more likely to require such monitoring in daily clinical practice. Addressing this gap, the present study aims to estimate the potential burden of TDM-requiring drug use in a NICU where routine TDM is not performed and to identify high-risk patient groups who may benefit from targeted monitoring strategies.

## Methods

### Study design and participants

This retrospective, descriptive study was conducted in the NICU of a tertiary-level teaching and research hospital in Türkiye. The unit consists of 28 beds (12 Level III and 16 Level II) and admits approximately 700 neonates annually, with around 90% of admissions being inborn. It primarily serves preterm and term neonates with medical conditions, while surgical cases are referred to specialized centers. All neonates hospitalized in the NICU between January 1, 2019, and July 1, 2024 (66 months), were screened, and those who received at least one medication requiring TDM were included in the retrospective analysis.

### Inclusion and exclusion criteria

The study included patients with a ≥ 24-hour NICU stay, aged 0–28 days, and who used at least one drug requiring TDM during their NICU hospitalization. Patients with missing or incomplete data were excluded from the study.

### Data collection

The patients’ sociodemographic information, birth weight, gestational age, admission diagnoses, drug treatments, mechanical ventilation (MV) status, total parenteral nutrition (TPN) status, discharge status, and mortality were obtained from the hospital information management system. Length of stay was defined as the duration of hospitalization in the NICU only. To ensure consistency, data extraction followed standardized protocols, and any discrepancies were resolved through consensus among the research team.

### Drugs requiring therapeutic drug monitoring

Drugs requiring TDM were identified based on established clinical guidelines and literature consensus [1–3, 5]. These included antibiotics (vancomycin, gentamicin, amikacin), antiepileptics (phenytoin, phenobarbital, carbamazepine, valproic acid, levetiracetam), and digoxin, all of which have narrow therapeutic windows and significant inter-individual pharmacokinetic variability.

### The primary outcomes of the study

The determination of the usage profile, frequency, and risk factors affecting the usage of drugs requiring TDM in the NICU.

### Sample size

Sample size calculation was based on annual NICU admission data. Approximately 700 patients were admitted to the NICU annually, and it was assumed that 50% of these patients used drugs requiring TDM. Using a power analysis with an alpha level of 0.05 and a power of 95%, the minimum required sample size for the study period was calculated to be 345 patients. Although the minimum required sample size was calculated as 345 patients, all eligible patients within the study period were included in the analysis to better reflect real-world clinical practice and increase the precision of the estimates.

### Statistical analysis

The statistical evaluation of the results was performed using SPSS 25.0 software. Chi-square analysis was applied to compare categorical data. For continuous variables, as their distribution was not normal (as assessed by the Shapiro-Wilk test), the non-parametric Mann-Whitney U test was used. Differences in the number of drugs requiring TDM across variables with more than two categories were tested using the Kruskal-Wallis test, followed by Tukey HSD post hoc analysis. Binary logistic regression analysis was performed to identify risk factors influencing the use of TDM-requiring drugs. For all analyses, *p*-values < 0.05 with a 95% confidence interval (CI) were considered statistically significant.

## Results

**Fig. 1:**
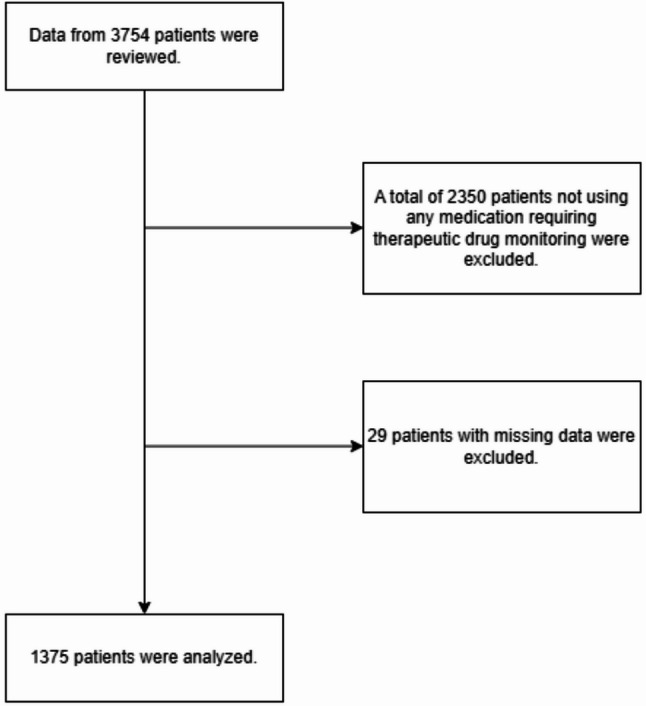
Study’s flowchart

A retrospective review identified 3754 patients, of whom 1404 (37.4%) received at least one medication requiring TDM. Among these, 1375 patients met the inclusion criteria and were included in the final analysis (Fig. 1). The majority of patients were male (68%), and the most common reasons for admission were respiratory distress (68.7%) and neonatal jaundice (10.7%). 58.2% of patients were followed in a level 3 NICU. Median [interquartile range (IQR)] gestational age was 36 weeks (33–37), 42% were classified as late preterm, and 32.7% were term. Median (IQR) birth weight was 2540 g (2025–2970), and 27.9% of patients were recorded as low birth weight (Table 1).

**Table 1:** Sociodemographic and clinical characteristics of patients

Variable	Total (*n* = 1375)
Sex, *n* (%)	
*Male*	935 (68)
*Female*	440 (32)
Causes of hospitalization, *n* (%)	
Respiratory distress	945 (68.7)
Neonatal jaundice	147 (10.7)
Other respiratory problems of the newborn	93 (6.8)
Other	190 (13.8)
Neonatal intensive care unit level, n (%)	
* Level 1*	72 (5.2)
* Level 2*	503 (36.6)
* Level 3*	800 (58.2)
Gestational age (weeks), median (IQR)	36 (33–37)
Gestational age, n (%) (week)	
* Extremely preterm (< 28)*	51 (3.7)
* Very preterm (28 to 32)*	157 (11.4)
* Moderate preterm (32 to 34)*	139 (10.1)
* Late preterm (34 to 37)*	578 (42)
* Term (≥ 37)*	450 (32.7)
Birth weight (g), median (IQR)	2540 (2025–2970)
Birth weight, n (%) (g)	
* Extremely low birth weight (< 1000)*	50 (3.6)
* Very low birth weight (1000 to 1500)*	103 (7.6)
* Low birth weight (1500 to 2500)*	384 (27.9)
* Normal birth weight (> 2500)*	838 (60.9)
Total NICU length of stay (day), median (IQR)	10 (6–22)
Discharge status, n (%)	
* Death*	28 (2)
* Discharged*	1347 (98)
Mechanical ventilation status, n (%)	
*Yes*	489 (35.6)
Duration of mechanical ventilation (day), median (IQR)	0 (0–2)
Total parenteral nutrition status n (%)	
* Yes*	470 (34.2)
Total parenteral nutrition duration (day), median (IQR)	0 (0–3)

*IQR* Interquartile range

**Fig. 2:**
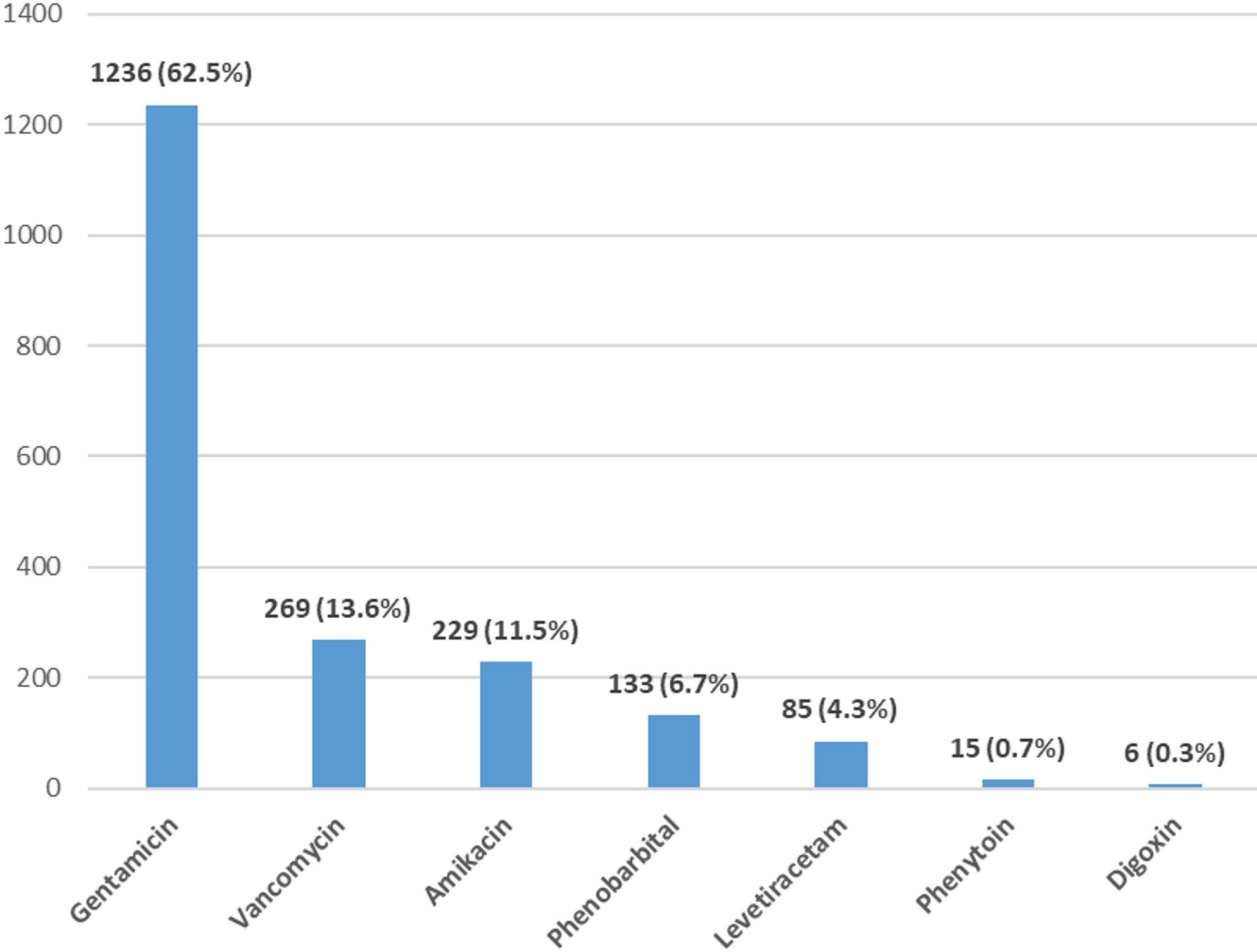
Frequency of medications requiring therapeutic drug monitoring

The median (IQR) of drugs requiring TDM was 1 (1–2), the mean ± standard deviation was 1.4 ± 0.8, and the minimum-maximum was 1–5. There were 1004 patients (73%) with one drug requiring TDM, 221 patients (16.1%) with two drugs, 91 patients (6.6%) with three drugs, 41 patients (3%) with four drugs and 18 patients (1.3%) with five drugs. Gentamicin (62.5%), vancomycin (13.6%), and amikacin (11.5%) were determined as the most common drugs requiring TDM in patients (Fig. 2).

There was no statistically significant difference between gender and gestational age categories and the total number of drugs requiring TDM (*p* > 0.05). However, MV status (*p* < 0.001), discharge status (*p* = 0.001), TPN status (*p* < 0.001), birth weight categories (*p* < 0.001), total number of days of NICU hospitalization (*p* < 0.001) and NICU level categories (*p* < 0.001) statistically significantly affect the total number of medications requiring TDM.

Univariate analyses exploring the factors associated with the number of medications requiring TDM are presented in Table 2. Level 3 NICU [odds ratio (OR) = 2.1, 95% CI = 1.6–2.8; *p* < 0.001], < 2500 g birth weight (OR = 2.6, 95% CI = 2–3.4; *p* < 0.001), presence of MV (OR = 4.3, 95% CI = 3.2–5.5; *p* < 0.001), presence TPN (OR = 4, 95% CI = 3.1–5.2; *p* < 0.001), and ≥ 10 days of NICU hospitalization (OR = 7.3, 95% CI = 5.4–9.9; *p* < 0.001) were associated with the increased likelihood of requiring TDM. Death was also associated with therapeutic drug monitoring (OR = 2.7, 95% CI = 1.3–5.8; *p* = 0.008).

**Table 2:** Statistical analysis of factors associated with the number of medications requiring therapeutic drug monitoring

Variables	Univariate analysis		Multivariate analysis	
	OR (95% CI)	*p*	OR (95% CI)	*p*
Level 3 NICU	2.1 (1.6–2.8)	< 0.001	1 (0.7–1.4)	0.659
< 2500 g birth weight	2.6 (2–3.4)	< 0.001	1 (0.8–1.4)	0.580
< 37 weeks gestational age	1 (0.8–1.3)	0.659	1.1 (0.8–1.5)	0.295
Presence of mechanical ventilation	4.3 (3.2–5.5)	< 0.001	2 (1.4–2.7)	< 0.001
Presence of total parenteral nutrition	4 (3.1–5.2)	< 0.001	1.6 (1.2–2.2)	0.002
≥ 10 days of NICU hospitalization	7.3 (5.4–9.9)	< 0.001	4.7 (3.3–6.5)	< 0.001
Death	2.7 (1.3–5.8)	0.008	1.9 (0.8–4.6)	0.111
Male gender	1 (0.8–1.3)	0.767	0.9 (0.7–1.3)	0.977

*CI* Confidence interval, *NICU* Neonatal intensive care unit, *OR* Odds ratio

Binary logistic regression analysis for the factors associated with the number of medications requiring TDM is presented in Table 2. A binomial logistic regression was performed to ascertain the effects of various factors on the likelihood of requiring TDM. Presence of MV (OR = 2, 95% CI = 1.4–2.7; *p* < 0.001), presence of TPN (OR = 1.6, 95% CI = 1.2–2.2; *p* = 0.002), and ≥ 10 days of NICU hospitalization (OR = 4.7, 95% CI = 3.3–6.5; *p* < 0.001) were independently associated with an increased likelihood of requiring TDM.

The logistic regression model was statistically significant, χ2 (6) = 276.4, *p* < 0.001. The model explained 26.5% (Nagelkerke R2 = 0.265) of the variance in medications requiring TDM and correctly classified 76.4% of cases. Sensitivity was 42%, specificity was 89.1%, positive predictive value was 58.9%, and negative predictive value was 80.6%. Of the eight predictor variables, only three were statistically significant: the presence of MV, the presence of TPN, and ≥ 10 days of NICU hospitalization (Table 2).

## Discussion

This study evaluated the prevalence, profile, and risk factors of drugs requiring TDM in NICU patients. The results revealed that one-third of patients required at least one TDM-requiring medication, with the most significant predictors being MV, TPN, and prolonged NICU hospitalization. These findings highlight the critical role of TDM in optimizing drug therapy in this vulnerable population. It should be noted that the outcome reflects the burden of exposure to TDM-requiring medications rather than their clinical effectiveness.

### Prevalence and profile of drugs requiring therapeutic drug monitoring

TDM is essential in improving treatment efficacy and minimizing toxicity in NICUs. This study found that 37.4% of NICU patients required TDM, consistent with the frequent use of aminoglycosides and antiepileptic drugs in neonatal treatment protocols [17–24]. Gentamicin was the most commonly prescribed TDM-requiring antibiotic (62.5%), while phenobarbital was the most common antiepileptic drug. These findings align with European NICU studies, where antibiotics are among the most frequently prescribed drugs [10]. Similarly, the high prevalence of phenobarbital and phenytoin use reflects their central role in managing neonatal seizures [16]. Notably, the increasing use of levetiracetam, with its favorable safety profile, was also evident in this study [14].

Gentamicin’s widespread use underscores its importance in treating gram-negative bacterial infections in neonates. However, its nephrotoxic potential necessitates precise dosing and TDM to minimize adverse effects [12, 25–27]. Interindividual variability in drug response, influenced by gestational age, birth weight, and organ maturity, highlights the need for individualized pharmacokinetic models in neonates [9].

### Risk factors for drug use requiring therapeutic drug monitoring

The study identified MV support, TPN use, and prolonged NICU hospitalization (≥ 10 days) as strong predictors of TDM-requiring drug use. These findings align with prior studies linking polypharmacy and critical illness to increased TDM requirements [9].

Low birth weight (< 2500 g) and admission to a level 3 NICU emerged as significant risk factors, highlighting the heightened fragility and more severe clinical conditions often observed in these patient groups [2]. Reduced drug clearance in neonates with immature renal and hepatic function contributes to higher toxicity risks, emphasizing the importance of TDM in these patients [6]. Furthermore, the association between mortality and TDM use (OR = 2.7) highlights the need for close monitoring in the most fragile neonates to prevent potentially fatal outcomes [13].

Premature neonates are particularly at risk due to ongoing nephrogenesis and exposure to nephrotoxic drugs such as aminoglycosides [28]. The delayed clearance of drugs like amikacin in extremely low birth weight (ELBW) neonates increases the risk of ototoxicity, necessitating careful dose adjustments based on gestational age, birth weight, and renal function [29–31]. Similarly, the immaturity of liver metabolism impacts the pharmacokinetics of drugs like phenobarbital, with premature neonates exhibiting longer half-lives and higher volumes of distribution compared to term neonates [32]. Therefore, monitoring medications that require TDM is essential for neonates presenting with the risk factors identified in the literature. However, it should be noted that routine implementation of TDM may not be feasible in all NICU settings, particularly in low- and middle-income countries. In such contexts, identifying high-risk patient groups may help prioritize limited resources and guide targeted monitoring strategies.

### Limitations and strengths

This study has several notable limitations. First, the retrospective design inherently limits causal inference, and the observed associations may partly reflect confounding by indication rather than direct causal relationships. Although significant associations were identified, prospective studies are required to confirm and further explore these findings. Second, the distribution of the outcome variable, with most patients receiving only a single TDM-requiring medication, may have limited variability and attenuated the strength of associations; however, even single-drug exposure remains clinically relevant in neonatal populations. Third, the absence of recorded laboratory data for TDM-requiring medications prevented an in-depth evaluation of whether drug concentrations achieved therapeutic targets. Importantly, this reflects real-world practice in a setting where routine TDM is not implemented. Consequently, data on plasma drug concentrations, dose adjustments, and TDM-related clinical outcomes were not available.

In addition, several potential confounding variables—including maternal risk factors, delivery room resuscitation, perinatal asphyxia, acute renal failure, and invasive procedures—could not be included due to incomplete or unavailable data in the hospital records. As a single-center study, the findings may also have limited generalizability to NICUs with different clinical practices and patient populations. Overall, the findings should be interpreted as descriptive and hypothesis-generating, providing a basis for future prospective studies aimed at evaluating the clinical impact of TDM in neonatal populations.

This study contributes to the limited body of literature by providing real-world data on the prevalence, profile, and risk factors associated with TDM-requiring medications in the NICU. The focus on a highly vulnerable and under-researched population enhances its clinical relevance. The relatively large sample size and robust statistical analyses offer valuable insights into key determinants of TDM-requiring drug use, including mechanical ventilation, total parenteral nutrition, and prolonged NICU hospitalization.

Future research should focus on prospective, multicenter studies to validate these findings and to evaluate the clinical impact of TDM implementation. Multidisciplinary collaboration remains essential for advancing safe and effective pharmacotherapy in NICUs.

## Conclusion

This study demonstrates that a substantial proportion of neonates in the NICU are exposed to medications requiring TDM. In a setting where routine TDM is not currently implemented, these findings highlight a potentially unmet need for monitoring. Mechanical ventilation, total parenteral nutrition, and prolonged NICU hospitalization were identified as key risk factors associated with TDM-requiring drug use. Identifying these high-risk groups may help guide targeted monitoring strategies and optimize resource allocation in NICUs where TDM is not routinely available.

## Data Availability

The datasets used and/or analysed during the current study are available from the corresponding author upon reasonable request.

## Abbreviations

AEDs: Antiepileptic Drugs
CI: Confidence Interval
ELBW: Extremely Low Birth Weight
IQR: Interquartile Range
MV: Mechanical Ventilation
NICU: Neonatal Intensive Care Unit
TDM: Therapeutic Drug Monitoring
TPN: Total Parenteral Nutrition
Vd: Volume of Distribution
